# Prediagnostic Serum Organochlorine Concentrations and Metastatic Prostate Cancer: A Nested Case–Control Study in the Norwegian Janus Serum Bank Cohort

**DOI:** 10.1289/ehp.1408245

**Published:** 2015-03-03

**Authors:** Stella Koutros, Hilde Langseth, Tom K. Grimsrud, Dana Boyd Barr, Roel Vermeulen, Lützen Portengen, Sholom Wacholder, Laura E. Beane Freeman, Aaron Blair, Richard B. Hayes, Nathaniel Rothman, Lawrence S. Engel

**Affiliations:** 1 Division of Cancer Epidemiology and Genetics, National Cancer Institute, National Institutes of Health, Department of Health and Human Services, Bethesda, Maryland, USA; 2 Department of Research, Cancer Registry of Norway, Institute of Population-Based Cancer Research, Oslo, Norway; 3 Department of Environmental Health, Rollins School of Public Health, Emory University, Atlanta, Georgia, USA; 4 Division of Environmental Epidemiology, Institute for Risk Assessment Sciences, Utrecht University, Utrecht, the Netherlands; 5 Division of Epidemiology, Department of Population Health, New York University School of Medicine, New York, New York, USA; 6 Department of Epidemiology, UNC Gillings School of Global Public Health, Chapel Hill, North Carolina, USA

## Abstract

**Background:**

Organochlorine (OC) insecticides and polychlorinated biphenyls (PCBs) have been shown to have estrogenic, antiestrogenic, or antiandrogenic properties; as a result, the impact of exposure to these compounds and risk of hormonal cancers, such as prostate cancer, is a concern.

**Objectives:**

We conducted a nested case–control study, using prospectively collected serum, to estimate associations between OC exposures and metastatic prostate cancer in a population-based cohort from Norway.

**Methods:**

Sera from 150 cases and 314 controls matched on date of blood draw, age at blood draw, and region was used to determine concentrations of 11 OC pesticide metabolites and 34 PCB congeners. Odds ratios (ORs) and 95% confidence intervals (95% CIs) for quartiles of lipid-corrected metabolite levels were calculated using conditional logistic regression.

**Results:**

Metastatic prostate cancer was two times as likely among men with serum concentrations of oxychlordane in the highest quartile compared with those in the lowest quartile (OR = 2.03; 95% CI: 1.03, 4.03; *p*-trend 0.05). Elevated but nonsignificant ORs were estimated for the highest versus lowest quartile of heptachlor epoxide, HCB, and mirex, although these exposures were correlated with oxychlordane. Findings for specific PCB congeners showed a significant inverse association between natural log–transformed lipid-adjusted PCB 44 and metastatic prostate cancer (OR = 0.74; 95% CI: 0.56, 0.97; *p*-trend = 0.02).

**Conclusions:**

Our study highlights the importance of estimating associations with specific OC chemicals and suggests a possible role of OC insecticides and PCBs in the etiology of metastatic prostate cancer.

**Citation:**

Koutros S, Langseth H, Grimsrud TK, Barr DB, Vermeulen R, Portengen L, Wacholder S, Beane Freeman LE, Blair A, Hayes RB, Rothman N, Engel LS. 2015. Prediagnostic serum organochlorine concentrations and metastatic prostate cancer: a nested case–control study in the Norwegian Janus Serum Bank cohort. Environ Health Perspect 123:867–872; http://dx.doi.org/10.1289/ehp.1408245

## Introduction

Organochlorines (OCs) are a diverse group of persistent synthetic compounds that have been used as pesticides (mostly insecticides) and for various industrial and commercial applications.

OC insecticides were widely used in agriculture and pest control between the 1940s and 1960s. Another group of OC compounds, polychlorinated biphenyls (PCBs), were historically used in numerous construction materials including plasticizers, adhesives, flame retardants, caulk, sealants, and paints, and in electrical equipment. These compounds resist degradation, bioaccumulate in adipose tissue of humans and other tissues, and persist in the environment. Many countries banned their production in the 1970s and 1980s, citing public health concerns (Longnecker et al. 1997). Evidence suggests that some of these OC compounds may cause a variety of adverse health effects, including cancer. The International Agency for Research on Cancer (IARC) lists PCBs as carcinogenic to humans (Group 1) (Lauby-Secretan et al. 2013) although the assessment for the OC insecticides is less clear, with some specific insecticides listed as possible human carcinogens (Group 2B), and others listed as not classifiable (Group 3) (IARC 1991).

The mechanisms by which OC compounds might influence cancer development are not completely understood; however, many of the chemicals have been shown to have estrogenic, antiestrogenic, or antiandrogenic properties (IARC 2012; Longnecker et al. 1997). Because of this, the impact of exposure to these compounds on risk of hormonal cancers, such as prostate cancer, is a concern. Several studies have reported associations between exposures to these compounds and prostate cancer among occupationally exposed populations (Prince et al. 2006; Purdue et al. 2007; Van Maele-Fabry and Willems 2004), but there is less information about associations with environmental exposures in the general population, which occur mainly from meat, fish, and dairy consumption (Kvalem et al. 2009; Schecter et al. 2010). Associations of environmental OC exposures and prostate cancer have been evaluated in eight epidemiologic studies (one ecologic study, one cross-sectional study, and six case–control studies) (Aronson et al. 2010; Hardell et al. 2006; Kumar et al. 2010; Multigner et al. 2010; Pavuk et al. 2004; Ritchie et al. 2003, 2005; Sawada et al. 2010; Xu et al. 2010). A small hospital-based case–control study from the United States reported positive associations of PCB congener 180 and the chlordane metabolite oxychlordane with prostate cancer (Ritchie et al. 2003). Another hospital-based study from Sweden showed significant positive associations between PCB 153 and the chlordane constituent *trans*-chlordane and prostate cancer (Hardell et al. 2006). Two additional studies have shown statistically significant positive associations of other OC insecticides, including chlordecone, β-hexachlorocyclohexane (HCH), and γ-HCH with prostate cancer (Kumar et al. 2010; Multigner et al. 2010). Conversely, two studies, including the only study to use prospectively collected serum, have reported inverse associations of prostate cancer with total PCBs overall, certain PCB congeners, and the OC insecticides β-HCH and γ-HCH (Aronson et al. 2010; Sawada et al. 2010).

Given these mixed results, we conducted a nested case–control study, using serum collected prior to diagnosis, to evaluate associations between environmental OC exposures and metastatic prostate cancer in the population-based Janus Serum Bank cohort of Norway.

## Methods

*Study population*. The Janus Serum Bank cohort is a population-based research biobank dedicated to cancer studies. The cohort consists of almost 317,000 individuals with an average age at enrollment of 41 years. Participants were mainly recruited from county-based health surveys in the 1970s and 1980s. Approximately 10% of the cohort members were recruited from Red Cross blood donors 18–65 years of age living in Oslo, the capital of Norway, with samples collected in the time period 1973–2004 (Cancer Registry of Norway 2014). The Janus cohort was linked by national personal identification numbers to the Cancer Registry of Norway to identify new cases of prostate cancer. Concern about possible detection bias associated with prostate specific antigen (PSA) testing, which began in earnest around 1990 in Norway (Kvåle et al. 2007), led us to include only metastatic prostate cancer, which is less likely to be identified by screening. Thus, for the current nested case–control study, eligible cases consisted of all 184 incident metastatic prostate cancer cases with no history of cancer (except nonmelanoma skin cancer), who were diagnosed from enrollment through 31 December 1999 and were diagnosed at least 2 years after serum collection. Metastasis and histologic grade were characterized according to the American Cancer Society’s *Manual of Tumor Nomenclature and Coding* (Percy et al. 1968) or the *International Classification of Diseases for Oncology* (ICD-O) (World Health Organization 1976), depending on the year of diagnosis. Controls were randomly selected male members of the cohort who had no history of cancer (except for nonmelanoma skin cancer) at the time of their matched case’s diagnosis. The present study was part of a series of case–control studies relating serum OC concentrations to multiple cancer end points within the Janus cohort. Initially, at least 1 control was matched to each prostate cancer case. To increase statistical power, controls initially selected for other end points also were included in the present analysis, allowing for up to 6 matched controls for some prostate cancer cases. A total of 389 eligible controls were identified for the prostate nested case–control study. Cases and controls were matched on date of blood draw (1-year strata), age at blood draw (2-year strata), and region (Finnmark, Oslo, Sogn og Fjordane, or Oppland). Sera from cases and their matched controls, including the augmented controls, were analyzed together in the same laboratory batch. Demographic data and other covariates [including body mass index (BMI) and smoking habits] were obtained from baseline questionnaire data at the National Institute of Public Health, and census data were obtained from Statistics Norway (Langseth et al. 2010). This project was approved by the regional committees for medical and health research ethics, and all subjects provided consent prior to the study.

*Laboratory analyses*. Concentrations of 11 OC pesticides or their metabolites [β-HCH, γ-HCH, dieldrin, hexachlorobenzene (HCB), mirex, *o,p´*-dichlorodiphenyltrichloroethane (DDT), *p,p´*-dichlorodiphenyldichloroethylene (DDE), *p,p´*-DDT, heptachlor epoxide, oxychlordane, and *trans*-nonachlor], 34 PCB congeners (PCBs 18, 28, 44, 49, 52, 66, 74, 87, 99, 101, 118, 128, 138, 146, 149, 151, 153, 156, 157, 167, 170, 172, 177, 178, 180, 183, 187, 189, 194, 195, 196, 201, 206, and 209), and lipid levels were measured at the Centers for Disease Control and Prevention, National Center for Environmental Health, in 0.8 mL of serum per participant available for the study. Measurements, methods, and quality control (QC) procedures for the larger study of OCs in relation to multiple cancer sites within the Janus cohort have been previously described in detail (Engel et al. 2007; Purdue et al. 2009). Briefly, serum samples were first spiked with isotopically labeled internal standards and then purified via automated accelerated solvent extraction and high-resolution gel permeation chromatography on a high-performance liquid chromatograph. Concentrated extracts were analyzed by gas chromatography/high-resolution mass spectrometry. Selected PCB congeners and OC pesticides in each sample were quantified from ^13^C isotope dilution continuing calibration plots, which automatically corrected for extraction efficiency. Values below the instrumental limits of detection (LOD) were imputed using a parametric model–based estimation procedure (Lubin et al. 2004). Using measurements among controls for a target analyte, we used maximum-likelihood methods to estimate parameters for the log-normal distribution. For each measurement < LOD, we randomly sampled a value from the appropriate log-normal distribution as the imputed value. As a QC check for possible errors in measurement due to interfering compounds, the ratio of ^35^Cl to ^37^Cl was calculated for each analyte and compared with the expected ratio for that analyte. Analyte measurements with observed ion ratios (IRs) greater than ± 20% of the expected IR were flagged as being out of tolerance and were recoded based on their proximity to the LOD: Flagged values ≤ 10 times the instrumental LOD were treated as if they were < LOD and imputed as described above, whereas flagged values > 10 times the instrumental LOD were recoded as missing data. Analytes with measurements either below the LOD or flagged as out of tolerance in at least half of the subjects (PCBs 87, 149, and 151) were also excluded from further analysis. Total lipid concentration was calculated for each subject using measurements of total cholesterol and triglycerides. Masked QC samples, including single samples from a large pool and pairs of replicate samples, were interspersed among study samples to assess intrabatch and interbatch variability. The median intrabatch coefficient of variation (CV) was 6 (range, 3–16) and the median interbatch CV was 37 (range, 17–165).

The serum samples from 29 cases were not successfully analyzed, and data were excluded for 5 cases from laboratory batches with aberrant measurements for both QC and subject samples, leaving 150 cases of prostate cancer for analysis. Serum samples from 56 controls were not successfully analyzed, and data from 19 controls in the same aberrant batches as their 5 matched cases were excluded, leaving 314 controls for analysis.

*Statistical analysis*. Lipid-corrected serum concentrations were modeled continuously and natural log–transformed. They were also categorized into quartiles based on the distributions of each analyte among controls, with the lowest quartile used as the reference category in analyses. We used conditional logistic regression matched on date of blood draw, age at blood draw, and region to calculate odds ratios (ORs) and 95% confidence intervals (95% CIs), and used the MIANALYZE procedure in SAS, version 9.2 (SAS Institute Inc., Cary, NC, USA), to obtain the appropriate variance for imputed data. Analyses were conducted for individual analytes, total chlordane/heptachlor–related compounds (sum of heptachlor epoxide, oxychlordane, and *trans*-nonachlor), and total PCBs. In addition, experimental evidence suggests that some PCBs may exert estrogenic and possibly antiandrogenic effects and induce cytochrome p450 activity (McFarland and Clarke 1989; Wolff et al. 1997). Thus, we also considered *a priori* groupings of PCB congeners based on previously suggested groupings (low chlorinated: PCBs 11, 18, 28, 44, 49, 52, 66, and 74; moderately chlorinated: PCBs 99, 105, 110, 118, 128, 138, 146, 153, 156, 157, 167, 170, 172, 177, 178, 180, 183, 187, and 189; highly chlorinated: PCBs 194, 195, 196, 201, 206, and 209; Wolff 1A: PCBs 44, 49, and 52; Wolff 1B: PCBs 177, 187, and 201; Wolff 2A: PCBs 66, 74, 105, 118, 156, and 167; Wolff 2B: PCBs 128, 138, and 170; Wolff 3: PCBs 99, 153, 180, 183, and 196). We also explored potential nonlinearity effects using 5-knot regression splines (piecewise polynomials). Likelihood ratio tests comparing the linear and spline models showed no improvement in model fit for the nonlinear model (data not shown), suggesting that the linear model, using the above defined quartiles, adequately fit the data. Separate analyses were conducted by grade of prostate cancer (moderately differentiated and poorly differentiated), time from blood draw to diagnosis (median: < 20 and ≥ 20 years), age at blood draw (median: ≤ 44 and > 44 years), BMI (< 25 kg/m^2^ and 25–29 kg/m^2^), as well as with additional adjustment for BMI (< 25 kg/m^2^, 25–29 kg/m^2^, ≥ 30 kg/m^2^) and smoking (never, former, current, missing). Likelihood ratio tests comparing models with and without the interaction term were used to formally assess differences between strata (*p*-interaction). Sensitivity analyses, excluding cases diagnosed after 1990 (when PSA testing began in earnest in Norway) and their matched controls, were also performed to assess the impact of detection bias. Tests for trend used the midpoint value of each exposure category treated as a continuous variable in regression models. All tests were two-sided and conducted at the α = 0.05 level.

## Results

The mean age at enrollment for prostate cancer cases was slightly higher (43.8 years) compared with controls (42.0 years) (Table 1). Year of enrollment, BMI, and cigarette smoking status were comparable between cases and controls. The mean age at diagnosis for cases was 63.1 years and, as expected for metastatic cancer, most of the tumors were moderately or poorly differentiated. Median concentrations and ranges of each analyte (including imputed values for measurements below the LOD) in cases and controls, as well as intrabatch and interbatch CVs and the percentage of measurements below the LOD of all OCs in the Janus prostate study, are presented in Supplemental Material, Table S1.

**Table 1:** Characteristics of prostate cases and controls in the Janus cohort.

Characteristic	Cases (*n *= 150)	Controls (*n *= 314)
Age at enrollment (mean ± SD)	43.8 ± 3.8	42.0 ± 6.2
Year of enrollment/collection [*n* (%)]
1972–1974	78 (52.0)	171 (54.5)
1975–1978	72 (48.0)	143 (45.5)
Body mass index [kg/m^2^; *n* (%)]
< 25	83 (55.3)	169 (53.8)
25–29	60 (40.0)	130 (41.4)
≥ 30	7 (4.7)	15 (4.8)
Cigarette smoking status [*n* (%)]
Never	26 (17.3)	58 (18.5)
Former	36 (24.0)	63 (20.1)
Current	73 (48.7)	163 (51.9)
Missing	15 (10.0)	30 (9.6)
Age at diagnosis (mean ± SD)	63.1 ± 5.3	—
Years from blood draw to diagnosis (mean ± SD)	17.8 ± 6.7	20.3 ± 4.9^*a*^
Tumor grade [*n* (%)]
Well differentiated	5 (3.3)	—
Moderately differentiated	66 (44.0)	—
Poorly differentiated	48 (32.0)	—
Unknown	31 (20.7)	—
^***a***^Defined as time from blood draw to matched case’s diagnosis date.		

Table 2 shows the association between OC insecticide metabolites and risk of metastatic prostate cancer. Metastatic prostate cancer was two times as likely to occur among men with serum concentrations of oxychlordane in the highest quartile compared with those in the lowest quartile [OR for quartile 4 (Q4) vs. quartile 1 (Q1) = 2.03, 95% CI: 1.03, 4.03; *p*-trend 0.05]. A similar association was estimated for heptachlor epoxide when comparing the highest quartile with the lowest quartile, although this was not statistically significant (OR for Q4 vs. Q1 = 2.01, 95% CI: 0.98, 4.10). Elevated but nonsignificant ORs were also estimated for the highest versus lowest quartiles of HCB and mirex. Heptachlor epoxide, HCB, and mirex were moderately correlated with oxychlordane [Spearman *r* = 0.55, *r* = 0.43 and *r* = 0.49, respectively (see Supplemental Material, Table S2)], suggesting that the results for these analytes may not be independent. However, mutually adjusted ORs based on models that included oxychlordane, heptachlor epoxide, HCB, and mirex were unstable due to multicollinearity (data not shown). The entire Spearman correlation matrix for pesticides measured in Janus is presented in Supplemental Material, Table S2.

**Table 2:** Association between organochlorine insecticides and risk of metastatic prostate cancer in the Janus cohort.

Metabolite	Continuous	Q1	Q2 vs. Q1		Q3 vs. Q1		Q4 vs. Q1		*p*-Trend^*c*^
	OR^*a,b*^ (95% CI)	Ca/Co	Ca/Co	OR^*b*^ (95% CI)	Ca/Co	OR^*b*^ (95% CI)	Ca/Co	OR^*b*^ (95% CI)	
Chlordane	1.09 (0.96, 1.24)	31/77	33/76	1.01 (0.52, 1.94)	44/81	1.39 (0.73, 2.62)	42/80	1.65 (0.82, 3.34)	0.13
Oxychlordane	1.26 (0.97, 1.64)	30/79	36/78	1.35 (0.70, 2.59)	37/79	1.34 (0.70, 2.55)	46/78	2.03 (1.03, 4.03)	0.05
*trans*-Nonachlor	1.29 (0.80, 2.08)	39/79	31/78	0.79 (0.43, 1.44)	35/79	0.89 (0.49, 1.63)	45/78	1.38 (0.73, 2.61)	0.19
Heptachlor epoxide	1.08 (0.82, 1.42)	32/78	33/79	1.01 (0.50, 2.02)	32/78	1.19 (0.60, 2.36)	52/79	2.01 (0.98, 4.10)	0.05
DDT	0.98 (0.88, 1.10)	34/79	36/78	0.94 (0.50, 1.78)	43/79	1.07 (0.59, 1.94)	37/78	0.97 (0.50, 1.88)	0.99
*p,p’*-DDT	0.97 (0.69, 1.38)	39/79	35/78	0.86 (0.46, 1.60)	33/79	0.76 (0.39, 1.47)	43/78	1.01 (0.52, 1.99)	0.75
*p,p’*-DDE	0.96 (0.72, 1.29)	35/79	32/78	0.80 (0.42, 1.51)	47/79	1.23 (0.69, 2.21)	36/78	0.90 (0.47, 1.73)	0.99
*o,p’*-DDT	0.96 (0.71, 1.28)	42/79	34/78	0.75 (0.41, 1.38)	27/79	0.62 (0.33, 1.19)	47/78	0.99 (0.50, 1.97)	0.58
Hexachlorobenzene	1.21 (0.75, 1.97)	29/79	49/78	1.70 (0.90, 3.22)	34/79	1.35 (0.62, 2.93)	38/78	2.03 (0.86, 4.84)	0.22
γ‑Hexachlorocyclohexane	1.01 (0.81, 1.26)	41/79	33/78	0.79 (0.42, 1.47)	31/79	0.80 (0.41, 1.56)	45/78	1.10 (0.57, 2.12)	0.67
β‑Hexachlorocyclohexane	1.19 (0.72, 1.95)	34/78	37/79	1.05 (0.56, 1.97)	29/79	0.75 (0.38, 1.50)	50/78	1.44 (0.71, 2.94)	0.29
Dieldrin	1.07 (0.87, 1.31)	32/78	37/77	1.14 (0.61, 2.15)	30/79	0.92 (0.46, 1.84)	50/80	1.42 (0.73, 2.77)	0.29
Mirex	1.26 (0.96, 1.65)	36/78	35/79	1.01 (0.55, 1.86)	34/79	0.94 (0.50, 1.77)	44/78	1.73 (0.90, 3.31)	0.07
Abbreviations: Ca/Co, cases/controls; DDE, dichlorodiphenyldichloroethylene; DDT, dichlorodiphenyltrichloroethane; OR, odds ratio; Q, quartile. ^***a***^Per unit increase in natural log–transformed ng/g lipid. ^***b***^Adjusted for county, age at collection, and date at collection. ^***c***^Trend of median values across quartiles.									

Table 3 shows the association between selected PCB congeners and PCB groupings and metastatic prostate cancer in the Janus study. Associations for the remaining PCB congeners are presented in Supplemental Material, Table S3. There was a significant inverse association between natural log–transformed lipid-adjusted PCB 44 and risk of metastatic prostate cancer (OR = 0.74, 95% CI: 0.56, 0.97; *p*-trend = 0.02; Table 3). An inverse association was also estimated for Wolff grouping 1A (*p*-trend = 0.03); there were no other statistically significant trends between any PCB congener or PCB grouping and metastatic prostate cancer (Table 3; see also Supplemental Material, Table S3).

**Table 3:** Association between select PCB congeners and PCB groupings and risk of metastatic prostate cancer in the Janus study.

Metabolite	Continuous	Q1	Q2 vs. Q1		Q3 vs. Q1		Q4 vs. Q1		*p*-Trend^*c*^
	OR^*a,b*^ (95% CI)	Ca/Co	Ca/Co	OR^*b*^ (95% CI)	Ca/Co	OR^*b*^ (95% CI)	Ca/Co	OR^*b*^ (95% CI)	
Selected PCB congener^*d*^
44	0.74 (0.56, 0.97)	48/79	38/78	0.74 (0.41, 1.33)	39/79	0.67 (0.35, 1.28)	25/78	0.39 (0.17, 0.86)	0.02
101	0.79 (0.57, 1.09)	48/79	28/78	0.52 (0.29, 0.95)	45/79	0.84 (0.47, 1.49)	29/78	0.51 (0.25, 1.03)	0.16
153	0.92 (0.59, 1.43)	35/79	39/78	1.01 (0.57, 1.78)	42/79	1.04 (0.58, 1.85)	34/78	0.83 (0.44, 1.56)	0.56
180	0.64 (0.36, 1.13)	31/77	43/79	1.19 (0.66, 2.17)	48/80	1.38 (0.75, 2.54)	28/78	0.71 (0.36, 1.38)	0.25
189	0.90 (0.73, 1.12)	47/78	41/79	0.93 (0.43, 2.00)	33/79	0.72 (0.34, 1.54)	29/78	0.64 (0.30, 1.37)	0.18
Total PCBs^*e*^	0.99 (0.98, 1.01)	38/78	38/79	0.82 (0.46, 1.45)	34/77	0.82 (0.45, 1.50)	40/80	0.92 (0.50, 1.68)	0.88
Degree of chlorination^*f*^
Low	0.95 (0.89, 1.01)	44/78	37/79	0.58 (0.31, 1.09)	34/78	0.58 (0.29, 1.17)	35/79	0.59 (0.26, 1.32)	0.44
Moderate	1.00 (0.98, 1.02)	33/76	38/80	0.92 (0.51, 1.65)	44/80	1.22 (0.68, 2.21)	35/78	0.94 (0.51, 1.73)	0.99
High	0.99 (0.91, 1.08)	38/78	35/79	1.00 (0.57, 1.77)	45/79	1.18 (0.67, 2.06)	32/78	0.89 (0.48, 1.64)	0.73
Wolff group^*g*^
1	0.95 (0.88, 1.02)	42/76	39/81	0.88 (0.49, 1.56)	41/78	0.97 (0.54, 1.72)	28/79	0.63 (0.32, 1.23)	0.19
1A	0.91 (0.81, 1.01)	48/78	36/79	0.67 (0.37, 1.19)	40/79	0.62 (0.33, 1.18)	26/78	0.39 (0.18, 0.85)	0.03
1B	0.97 (0.86, 1.10)	38/78	38/79	0.92 (0.52, 1.63)	39/78	1.06 (0.60, 1.90)	35/79	0.88 (0.48, 1.62)	0.77
2	0.99 (0.93, 1.05)	38/77	35/80	0.80 (0.44, 1.43)	41/78	1.09 (0.62, 1.91)	36/79	0.85 (0.45, 1.60)	0.85
2A	0.99 (0.90, 1.09)	40/78	38/78	0.84 (0.47, 1.50)	35/80	0.89 (0.50, 1.60)	37/78	0.91 (0.48, 1.72)	0.88
2B	0.98 (0.87, 1.10)	33/78	42/78	1.14 (0.64, 2.03)	43/79	1.23 (0.69, 2.19)	32/79	0.85 (0.46, 1.60)	0.60
3	0.96 (0.87, 1.06)	34/78	40/79	1.06 (0.60, 1.86)	43/79	1.13 (0.63, 2.05)	33/78	0.84 (0.45, 1.57)	0.57
Abbreviations: Ca/Co, cases/controls; OR, odds ratio; PCB, polychlorinated biphenyl; Q, quartile. ^***a***^Per unit increase in natural log–transformed ng/g lipid. ^***b***^Adjusted for county, age at collection, and date at collection. ^***c***^Trend of median values across quartiles. ^***d***^Estimates for the remaining PCBs are provided in Supplemental Material, Table S3. ^***e***^Sum of all congeners included in the analysis. ^***f***^Low: PCBs 11, 18, 28, 44, 49, 52, 66, and 74; moderate: PCBs 99, 105, 110, 118, 128, 138, 146, 153, 156, 157, 167, 170, 172, 177, 178, 180, 183, 187, and 189; high: PCBs 194, 195, 196, 201, 206, and 209. ^***g***^Wolff 1A: PCBs 44, 49, and 52; Wolff 1B: PCBs 177, 187, and 201; Wolff 2A: PCBs 66, 74, 105, 118, 156, and 167; Wolff 2B: PCBs 128, 138, and 170; Wolff 3: PCBs 99, 153, 180, 183, and 196.									

Separate analyses conducted by grade of prostate cancer, time from blood draw to diagnosis, age at blood draw, and BMI showed no significant interactions and are therefore not shown. Additional adjustment for BMI and for smoking had only negligible effect on the observed OC–disease associations and are therefore also not shown. Sensitivity analyses, excluding cases diagnosed after 1990 (*n* = 40), also produced minimal differences in the observed associations (data not shown).

## Discussion

In this analysis, we estimated a significant positive association between metastatic prostate cancer and prediagnostic serum concentrations of the chlordane metabolite oxychlordane. Elevated, but nonstatistically significant, associations were also estimated for the chlordane/heptachlor-related metabolite heptachlor epoxide and two other OC insecticides, HCB and mirex. Conversely, there were largely null associations for PCB congeners and PCB groupings with the exception of PCB 44, which was associated with a significant inverse risk of metastatic prostate cancer. These data highlight the importance of looking at specific OC chemicals in evaluating risk and suggest a possible role of OC insecticides and PCBs in the etiology of metastatic prostate cancer.

Chlordane and heptachlor are structurally related OC insecticides that have been considered together in toxicity and carcinogenicity evaluations because the technical-grade product of each contained about 10–20% of the other compound (IARC 2001). Their use has currently been banned or severely restricted in many countries; however, they are environmentally persistent. IARC lists both of these compounds as possibly carcinogenic to humans (Group 2B) owing to their ability to induce DNA damage and generate reactive oxygen species in experimental animal models (IARC 2001). These mechanisms, as well as postulated estrogenic or antiandrogenic activity (Diamanti-Kandarakis et al. 2009), could play a role in prostate carcinogenesis. In the present study, metastatic prostate cancer was positively associated with the chlordane and heptachlor serum metabolites oxychlordane and heptachlor epoxide, particularly for the highest versus lowest quartiles of exposure. Elevated but nonsignificant ORs were also estimated for the highest versus lowest quartiles of HCB and mirex. Because serum concentrations of oxychlordane, heptachlor epoxide, HCB, and mirex are moderately correlated, it is difficult to estimate independent effects of these metabolites, although these results do suggest that some or all of these agents may be involved. Other studies of circulating OCs and prostate cancer were more likely to show positive associations between chlordane or chlordane metabolites (Hardell et al. 2006; Ritchie et al. 2003) and prostate cancer, but further data are needed to prove whether this insecticide or its metabolites are etiologically important. It is also possible that our findings might be due to chance given the large number of comparisons considered.

Previous studies have shown associations between PCB 180 and PCB 153 and prostate cancer (Hardell et al. 2006; Ritchie et al. 2003). These two congeners were measured with good accuracy and were also present in the highest concentrations in our study (see Supplemental Material, Table S1); however, they were not associated with metastatic prostate cancer in our study population. Results were null for all other congeners, with the exception of an inverse association between PCB 44 and metastatic prostate cancer. Two other studies have also reported inverse associations between specific PCB congeners and prostate cancer (Aronson et al. 2010; Sawada et al. 2010), including the only other study that used prospectively collected samples (Sawada et al. 2010). Although it is unexpected that PCBs might be protective for prostate cancer, it is possible that the inverse associations could be reflective of real hormonal perturbations associated with PCB exposure that might also decrease prostate cancer development. Thus, the observed inverse association we, and others, have observed deserves further attention.

Few studies have looked at OCs in relation to more aggressive or lethal forms of prostate cancer. Sawada et al. (2010) found no association between serum OC insecticide concentrations and advanced prostate cancer, but they found an inverse association between total PCB concentrations and advanced prostate cancer. Conversely, Multigner et al. (2010) estimated stronger associations of serum concentrations of the OC insecticide chlordecone with highly aggressive forms of prostate cancer compared with less aggressive forms. In the occupational setting, a significant association between exposure to the OC insecticide aldrin and aggressive prostate cancer was observed in U.S. farmers, while there was no association with nonaggressive disease (Koutros et al. 2013). It should be noted, however, that each of the above studies used different methods to characterize more advanced/aggressive forms of prostate cancer, making comparisons of results across studies more difficult to interpret. Because prostate cancer is present in a large proportion of older men as an indolent disease, exposures positively associated with metastatic, but not indolent, prostate cancer might suggest factors involved in the progression of early stage disease to the more readily detectable aggressive form. Future work on the mechanisms by which OC insecticides might impact the development of aggressive or lethal forms of prostate cancer would be valuable.

Our study is one of the largest to evaluate environmental OC serum concentrations and risk of prostate cancer and only the second to use prospectively collected samples. Also, samples were collected in the 1970s and likely reflect peak or near-peak body burden of these compounds among the general population. In fact, concentrations of most OC metabolites were generally higher in the Janus cohort compared with more contemporary studies of prostate cancer cases and controls from Japan, Sweden, Canada, and the United States (Aronson et al. 2010; Ritchie et al. 2003; Sawada et al. 2010; Xu et al. 2010). Identification of metastatic disease also allowed us to consider the more lethal form of prostate cancer and to reduce the possibility of detection bias due to PSA screening because metastatic cancer is usually not detected by screening and may have a different etiology than more slow growing, or nonaggressive forms of the disease (Giovannucci et al. 2007). Nonetheless, some limitations must be acknowledged. QC results showed that measurement precision for analytes was variable. Quartile analyses resulted in modest numbers of cases in some categories, limiting the power to detect associations if they exist, in particular for stratified analyses. Because this study, and other prospective studies, have shown modest estimated effects for the association between OC compounds and advanced forms of prostate cancer, future studies should try to improve on the power to detect modest associations (in the range of 1.5–2.0) with more aggressive forms of the disease. Also, the high degree of correlation between many of these compounds limited our ability to examine independent effects. The power to examine interactions between OC metabolites and several factors was limited and thus provided little additional insight into the possible modification of associations between OC exposures and metastatic prostate cancer. Given the large number of measured compounds and general borderline significance level of observed findings, correction for multiple comparisons would have eliminated all observed effects, so we cannot rule out the possibility of false-positive associations. We also cannot rule out the possibility of false-negative associations, which may be a consequence of exposure misclassification.

## Conclusions

Metastatic prostate cancer was positively associated with prediagnostic serum concentrations of the OC insecticide metabolites oxychlordane, heptachlor epoxide, HCB, and mirex. Findings for specific PCB congeners or groupings were largely null, although there was a significant inverse association with PCB 44. These results suggest that certain OC insecticides and PCB congeners may contribute to metastatic prostate cancer. Additional prospective epidemiologic studies with the ability to look at the more aggressive forms of prostate cancer are needed to identify etiologic factors associated with the more lethal form of this disease. These studies should consider whether individual OC compounds appear to influence disease, as associations appear to be chemical specific, and should consider both positive and inverse associations suggested by this and other studies.

## Supplemental Material

(257 KB) PDF
