# Supplementary material for: Prediagnostic Serum Organochlorine Concentrations and Metastatic Prostate Cancer: A Nested Case–Control Study in the Norwegian Janus Serum Bank Cohort
Source: Environ Health Perspect. 2015 Mar 3;123(9):867–72. doi: 10.1289/ehp.1408245 (PMC4559951; doi:10.1289/ehp.1408245)
Supplement: (257 KB) PDF [file ehp.1408245.s001.acco.pdf]

Environ Health Perspect
doi: 10.1289/ehp.1408245

Note to Readers: *EHP* strives to ensure that all journal content is accessible to all readers. However, some figures and Supplemental Material published in *EHP* articles may not conform to 508 standards due to the complexity of the information being presented. If you need assistance accessing journal content, please contact ehp508@niehs.nih.gov. Our staff will work with you to assess and meet your accessibility needs within 3 working days.

Supplemental Material

Prediagnostic Serum Organochlorine Concentrations and Metastatic Prostate Cancer: A Nested Case–Control Study in the Norwegian Janus Serum Bank Cohort

Stella Koutros, Hilde Langseth, Tom K. Grimsrud, Dana Boyd Barr, Roel Vermeulen, Lützen Portengen, Sholom Wacholder, Laura E. Beane Freeman, Aaron Blair, Richard B. Hayes, Nathaniel Rothman, and Lawrence S. Engel

Table of Contents

Table S1. Median concentration of pesticides and PCB congeners in the Janus prostate study.

Table S2. Spearman Correlation Matrix for Pesticides Measured in the Janus prostate study.

Table S3. Association between PCB congeners not shown in Table 3 and risk of prostate cancer in the JANUS prostate study.

Table S1. Median concentration of pesticides and PCB congeners in the Janus prostate study.

Analyte (ng/g lipid)	Cases n=150 Median (Range)	Controls n=314 Median (Range)	Intrabatch CV Median	Interbatch CV Median	% below Limit of detection
Chlordane	60.6 (12.5-605.4)	57.2 (5.1-2207.7)			
Oxychlordane	19.0 (0.9-190.8)	17.5 (0.57-87.7)	8	30	15
trans-Nonachlor	26.9 (8.2-284.3)	26.1 (4.0-161.7)	7	28	0
Heptachlor Epoxide	13.4 (0.1-130.3)	12.8 (0.3-2158.4)	7	112	9
DDT	2552.1 (20.9-29110.4)	2432.4 (7.3-44158.6)			
p,p'-DDT	288.2 (13.8-2120.3)	282.1 (0.9-2943.2)	4	21	0
p,p'-DDE	2232.1 (6.3-27170.1)	2071.8 (5.8-43636.7)	5	58	0
o,p'-DDT	21.9 (0.7-225.8)	22.1 (0.6-381.5)	7	165	1
Hexachlorobenzene	514.6 (51.0-3508.4)	559.8 (58.9-3185.9)	16	58	0
γ-Hexachlorocyclohexane	8.0 (0.5-4621.4)	7.8 (0.5-124.0)	8	56	24
β-Hexachlorocyclohexane	166.2 (15.0-2990.0)	158.7 (41.5-1249.1)	5	22	0
Dieldrin	63.2 (1.4-556.6)	58.3 (1.0-1820.4)	6	144	11
Mirex	1.8 (0.1-37.1)	1.7 (0.1-18.3)	11	33	12
PCB Congener					
18	42.2 (9.0-448.1)	44.8 (5.0-832.9)	6	42	0
28	82.5 (11.2-1015.1)	80.5 (13.2-1553.2)	4	38	0
44	10.9 (0.8-118.1)	12.2 (0.5-211.7)	6	37	5
49	6.6 (0.1-83.7)	7.4 (0.4-179.8)	6	40	2
52	19.8 (4.4-154.3)	21.5 (2.8-319.3)	5	34	0
66	18.5 (2.5-218.2)	18.9 (1.5-92.2)	6	29	0
74	50.6 (11.4-398.0)	50.7 (1.0-238.8)	7	24	0
99	70.1 (3.3-407.1)	70.0 (1.0-417.9)	7	28	0
101	14.6 (1.4-123.0)	14.7 (1.5-115.7)	7	34	4
118	108.0 (8.2-754.5)	106.4 (7.2-502.9)	3	24	0
128	3.6 (0.03-40.1)	3.3 (0.005-27.3)	8	45	40
138	187.1 (14.1-716.3)	189.1 (56.0-834.3)	5	21	0
146	49.3 (8.5-175.3)	49.7 (7.1-173.9)	5	62	0
153	405.5 (40.6-1421.9)	404.5 (1.6-1425.3)	4	20	0
156	32.0 (2.2-111.1)	33.0 (2.0-190.5)	4	20	0

Analyte (ng/g lipid)	Cases<br>n=150<br>Median (Range)	Controls<br>n=314<br>Median (Range)	Intrabatch CV Median	Interbatch CV Median	% below Limit of detection
157	5.7 (0.04-109.7)	5.3 (0.1-119.9)	6	90	14
167	9.5 (0.7-47.7)	9.6 (0.8-35.3)	8	30	13
170	91.7 (9.0-260.7)	94.9 (29.1-275.3)	4	19	0
172	12.8 (0.6-48.3)	12.1 (0.5-42.5)	8	129	10
177	17.7 (1.9-48.3)	18.0 (1.7-72.0)	6	21	0
178	12.6 (0.9-37.5)	12.7 (0.4-44.7)	3	47	13
180	209.5 (4.1-605.0)	211.8 (25.6-605.4)	5	20	0
183	28.4 (2.6-100.1)	28.5 (3.2-85.2)	5	21	0
187	69.8 (9.1-227.9)	70.7 (6.6-217.8)	6	18	0
189	2.1 (0.003-8.7)	2.8 (0.003-11.8)	13	52	32
194	26.6 (3.7-70.9)	27.2 (3.4-107.2)	6	17	0
195	7.1 (0.9-19.1)	7.2 (0.8-20.8)	10	31	4
196	13.9 (2.8-41.6)	14.1 (2.1-66.5)	7	24	0
201	22.6 (3.2-73.3)	23.0 (2.8-94.1)	7	18	0
206	9.8 (1.5-34.4)	9.7 (1.4-34.5)	5	17	0
209	8.1 (1.1-27.7)	7.6 (0.1-108.3)	4	18	0
Total PCBs	1710.4 (330.1-6069.8)	1763.2 (318.6-4836.8)			
Low Chlorinated	227.3 (63.7-1388.3)	246.4 (74.3-3363.7)			
Medium Chlorinated	1355.2 (151.5-4976.5)	1343.0 (257.8-4468.3)			
High Chlorinated	89.8 (20.4-240.0)	87.8 (10.9-366.6)			
Wolff 1	171.6 (32.1-544.3)	177.8 (44.7-839.4)			
Wolff 1a	37.9 (8.4-356.1)	40.3 (4.9-710.8)			
Wolff 1b	127.1 (17.3-361.1)	129.2 (27.6-426.9)			
Wolff 2	515.3 (57.9-2433.4)	513.2 (109.2-1594.8)			
Wolff 2a	220.0 (34.8-1431.2)	225.0 (13.4-813.3)			
Wolff 2b	280.3 (23.1-1002.2)	283.1 (95.7-1126.5)			
Wolff 3	732.8 (88.6-2518.3)	736.0 (111.9-2452.9)			
Total PCBs	1710.4 (330.1-6069.8)	1763.2 (318.6-4836.8)			

Table S2. Spearman Correlation Matrix for Pesticides Measured in the Janus prostate study.

Analyte	Chlordane	OXY	TNA	HPE	DDT	PDT	PDE	ODT	HCB	BHC	GHC	Dieldrin	Mirex
Chlordane	1	0.93	0.80	0.76	0.41	0.41	0.42	0.29	0.43	0.35	0.22	0.46	0.47
Oxychlordane	0.93	1	0.74	0.55	0.32	0.32	0.38	0.20	0.43	0.27	0.17	0.33	0.49
trans-Nonachlor	0.80	0.74	1	0.57	0.47	0.43	0.45	0.39	0.40	0.34	0.17	0.40	0.53
Heptachlor Epoxide	0.75	0.55	0.57	1	0.48	0.50	0.44	0.39	0.38	0.49	0.30	0.59	0.19
DDT	0.41	0.32	0.47	0.48	1	0.92	0.86	0.91	0.24	0.52	0.22	0.53	0.03
p,p'-DDT	0.41	0.32	0.43	0.50	0.92	1	0.72	0.80	0.23	0.53	0.26	0.56	-0.001
p,p'-DDE	0.42	0.38	0.45	0.44	0.86	0.73	1	0.65	0.30	0.47	0.15	0.36	0.11
o,p'-DDT	0.29	0.20	0.38	0.39	0.91	0.80	0.65	1	0.13	0.45	0.19	0.52	-0.03
Hexachlorobenzene	0.43	0.43	0.40	0.38	0.24	0.23	0.30	0.13	1	0.25	0.18	0.18	0.24
β-Hexachlorocyclohexane	0.35	0.27	0.34	0.49	0.52	0.53	0.47	0.44	0.25	1	0.23	0.51	-0.07
γ-Hexachlorocyclohexane	0.22	0.17	0.17	0.30	0.21	0.26	0.15	0.19	0.18	0.23	1	0.28	0.01
Dieldrin	0.47	0.33	0.40	0.59	0.53	0.56	0.36	0.52	0.18	0.51	0.28	1	0.04
Mirex	0.47	0.49	0.53	0.190	0.02	-0.001	0.11	-0.03	0.23	-0.07	0.01	0.04	1

Abbreviations: OXY: Oxychlordane; TNA: trans-Nonachlor; HPE: Heptachlor Epoxide; PDT: p,p'-DDT; PDE: p,p'-DDE; ODT: o,p'-DDT; HCB: Hexachlorobenzene; BHC: β-Hexachlorocyclohexane; GHC:γ-Hexachlorocyclohexane.

Table S3. Association between PCB congeners not shown in Table 3 and risk of prostate cancer in the JANUS prostate study.

PCB congener	Continuous OR[a,b] (95% CI)	Q2 vs. Q1 OR[b] (95% CI)	Q3 vs. Q1 OR[b] (95% CI)	Q4 vs. Q1 OR[b] (95% CI)
PCB 18	0.93 (0.65, 1.34)	2.22 (1.16, 4.24)	1.92 (0.92, 3.99)	1.02 (0.44, 2.38)
PCB 28	0.75 (0.49, 1.16)	0.81 (0.41, 1.61)	0.91 (0.43, 1.94)	0.81 (0.33, 2.00)
PCB 49	0.90 (0.70, 1.15)	0.99 (0.55, 1.78)	0.77 (0.39, 1.53)	0.49 (0.21, 1.14)
PCB 52	0.70 (0.48, 1.03)	0.77 (0.43, 1.37)	0.61 (0.32, 1.17)	0.55 (0.26, 1.16)
PCB 66	0.82 (0.55, 1.22)	0.38 (0.19, 0.73)	0.61 (0.33, 1.13)	0.64 (0.30, 1.34)
PCB 74	0.88 (0.57, 1.37)	0.71 (0.39, 1.30)	1.06 (0.58, 1.94)	0.69 (0.35, 1.34)
PCB 99	0.90 (0.61, 1.34)	1.22 (0.69, 2.16)	1.39 (0.77, 2.50)	0.91 (0.48, 1.75)
PCB 118	0.98 (0.61, 1.58)	1.00 (0.56, 1.80)	1.08 (0.60, 1.95)	1.07 (0.56, 2.02)
PCB 128	1.00 (0.87, 1.15)	0.71 (0.29, 1.76)	1.04 (0.55, 1.98)	0.99 (0.52, 1.89)
PCB 138	0.80 (0.46, 1.38)	1.38 (0.76, 2.52)	1.26 (0.69, 2.31)	1.12 (0.60, 2.10)
PCB 146	1.01 (0.65, 1.55)	1.20 (0.68, 2.12)	1.17 (0.64, 2.13)	1.08 (0.58, 2.01)
PCB 156	0.95 (0.61, 1.48)	1.14 (0.64, 2.03)	1.38 (0.77, 2.47)	0.80 (0.42, 1.52)
PCB 157	0.99 (0.80, 1.24)	0.82 (0.41, 1.66)	1.27 (0.69, 2.33)	0.86 (0.43, 1.71)
PCB 167	1.08 (0.83, 1.40)	0.89 (0.46, 1.72)	0.77 (0.41, 1.47)	1.23 (0.67, 2.29)
PCB 170	0.72 (0.41, 1.27)	1.13 (0.64, 1.99)	1.19 (0.67, 2.12)	0.76 (0.40, 1.43)
PCB 172	0.95 (0.74, 1.21)	0.84 (0.44, 1.60)	1.60 (0.88, 2.91)	0.81 (0.42, 1.56)
PCB 177	1.15 (0.80, 1.66)	1.45 (0.82, 2.57)	1.20 (0.65, 2.21)	0.95 (0.52, 1.73)
PCB 178	0.97 (0.75, 1.25)	1.14 (0.60, 2.18)	1.13 (0.61, 2.09)	0.94 (0.50, 1.77)
PCB 183	0.91 (0.64, 1.30)	1.29 (0.72, 2.29)	1.23 (0.69, 2.16)	0.90 (0.48, 1.67)
PCB 187	0.88 (0.54, 1.42)	0.99 (0.57, 1.75)	0.92 (0.51, 1.67)	0.92 (0.50, 1.70)
PCB 194	0.89 (0.54, 1.46)	1.09 (0.62, 1.94)	1.08 (0.61, 1.92)	0.86 (0.47, 1.58)
PCB 195	0.91 (0.65, 1.26)	1.23 (0.69, 2.21)	1.05 (0.58, 1.91)	0.82 (0.44, 1.50)
PCB 196	0.81 (0.47, 1.39)	1.21 (0.69, 2.14)	1.12 (0.64, 1.99)	0.71 (0.37, 1.36)
PCB 201	0.93 (0.59, 1.49)	1.07 (0.61, 1.90)	0.99 (0.56, 1.75)	0.85 (0.46, 1.60)
PCB 206	1.25 (0.75, 2.10)	0.82 (0.46, 1.46)	0.99 (0.56, 1.73)	0.95 (0.53, 1.72)
PCB 209	1.06 (0.68, 1.63)	0.95 (0.52, 1.74)	1.20 (0.67, 2.14)	1.04 (0.56, 1.94)

Abbreviations: OR: Odds Ratio; LCL: Lower 95% Confidence Limit; UCL: Upper 95% Confidence Limit.

[a]Per unit increase in natural log-transformed ng/g lipid. [b]Adjusted for county, age at collection, date at collection.
